# Induction, growth, drug resistance, and metastasis: A comprehensive summary of the relationship between STAT3 and gastric cancer

**DOI:** 10.1016/j.heliyon.2024.e37263

**Published:** 2024-09-04

**Authors:** Muyang Chen, Tongshan Wang, Dianzhe Tian, Chaorui Hai, Zixuan Qiu

**Affiliations:** a School of Pediatrics, Nanjing Medical University, Nanjing, China; b Gastric Cancer Center, Department of Oncology, the First Affiliated Hospital of Nanjing Medical University, Nanjing, China; c Chinese Academy of Medical Sciences and Peking Union Medical College, Beijing, China; d School of Life Sciences, Lanzhou University, Lanzhou, China; e School of Public Health, Xiangya School of Medicine, Central South University, Changsha, China

**Keywords:** STAT3, Gastric cancer, MicroRNA, Small molecule inhibitor, Signaling pathway

## Abstract

Gastric cancer is a prevalent and highly lethal malignancy that poses substantial challenges to healthcare systems globally. Owing to its often asymptomatic nature in early stages, diagnosis frequently occurs at advanced stages when surgical intervention is no longer a viable option, forcing most patients to rely on nonsurgical treatments such as chemotherapy, targeted therapies, and emerging immunotherapies. Unfortunately, the therapeutic response rates for these treatments are suboptimal, and even among responders, the eventual development of drug resistance remains a significant clinical hurdle. Signal transducer and activator of transcription 3 (STAT3) is a widely expressed cellular protein that plays crucial roles in regulating cellular processes such as growth, metabolism, and immune function. Aberrant activation of the STAT3 pathway has been implicated in the initiation, progression, and therapeutic resistance of several cancers, with gastric cancer being particularly affected. Dysregulated STAT3 signaling not only drives tumorigenesis but also facilitates the development of resistance to chemotherapy and targeted therapies, as well as promotes metastatic dissemination. In this study, we explored the critical role of the STAT3 signaling cascade in the pathogenesis of gastric cancer, its contribution to drug resistance, and its involvement in the metastatic process. Furthermore, we assess recent advances in the development of STAT3 inhibitors and their potential application as therapeutic agents in the treatment of gastric cancer. This work provides a comprehensive overview of the current understanding of STAT3 in gastric cancer and offers a foundation for future research aimed at improving therapeutic outcomes in this challenging disease.

## 1 Introduction

**Fig. 1:**
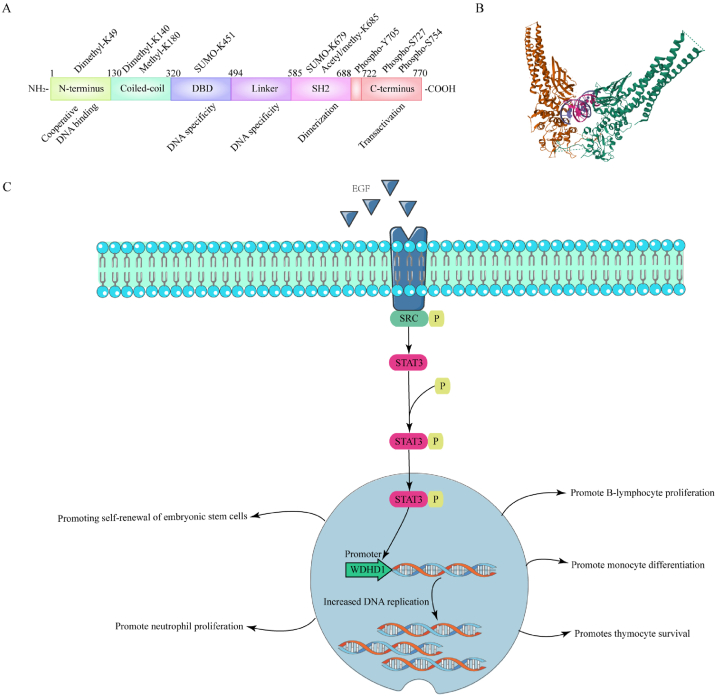
Basic structure of STAT3.

**Fig. 2:**
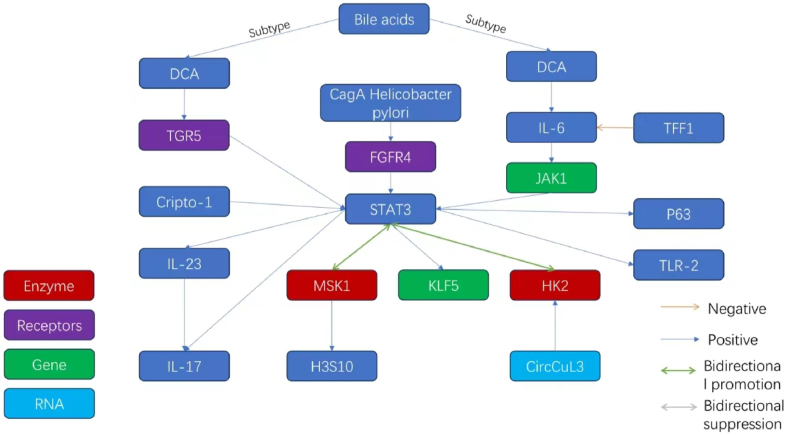
The molecular network of STAT3 and the induction of gastric cancer.

**Fig. 3:**
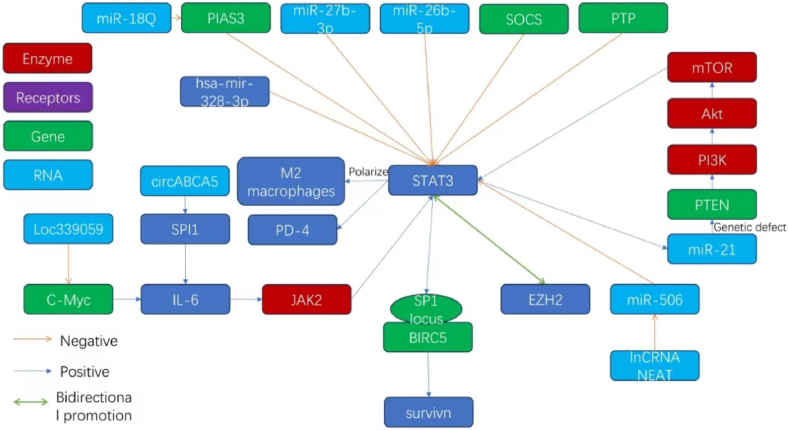
The molecular network of STAT3 and the growth and progression of gastric cancer (1).

**Fig. 4:**
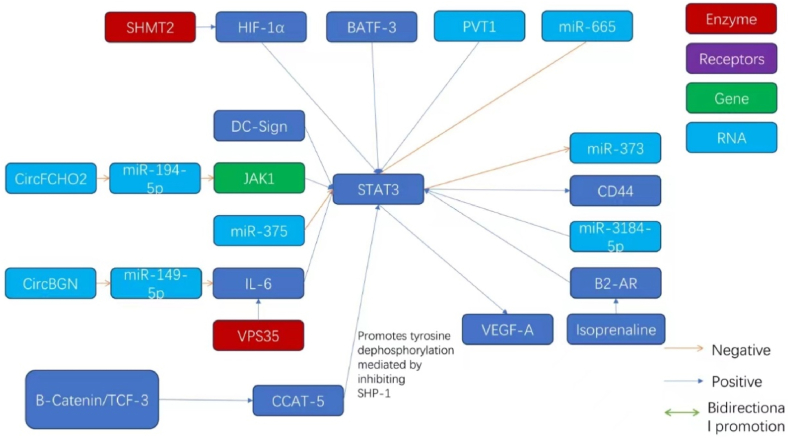
The molecular network of STAT3 and the growth and progression of gastric cancer (2).

**Fig. 5:**
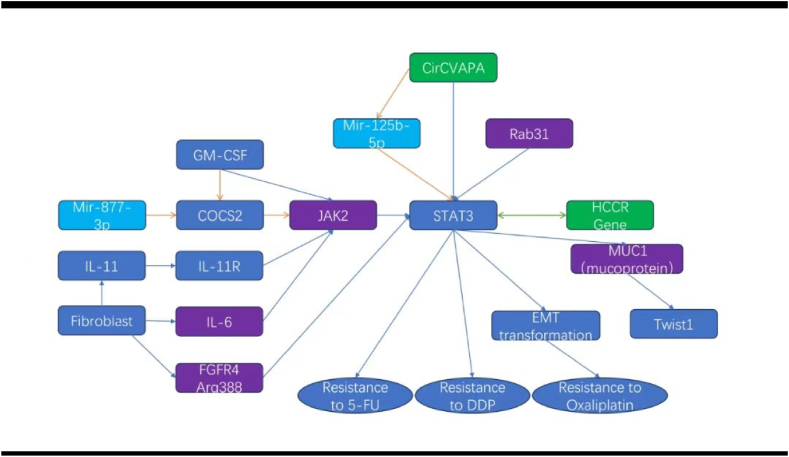
A molecular interaction network related to STAT3 and resistance of gastric cancer to chemotherapy.

**Fig. 6:**
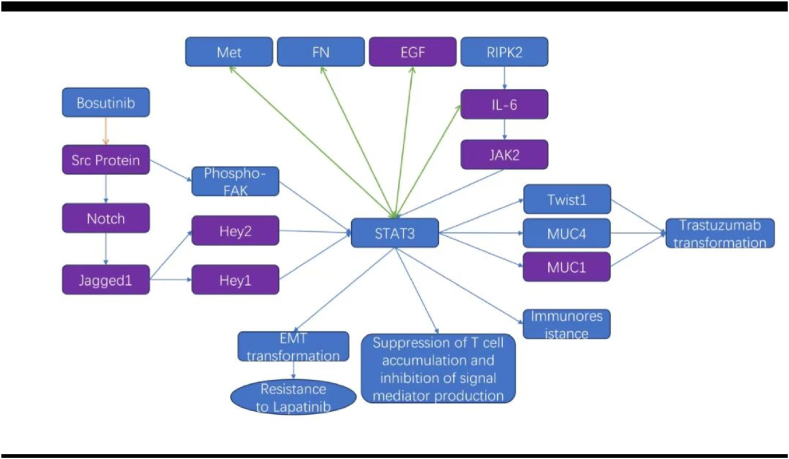
Molecular interaction network related to STAT3, gastric cancer targeting, and immune resistance.

**Fig. 7:**
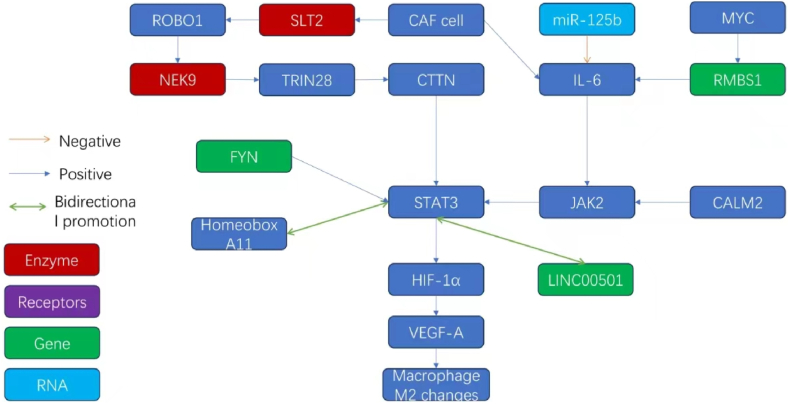
Molecular interaction network related to STAT3 and gastric cancer metastasis.

Gastric carcinoma ranks as the fifth most prevalent cancer and the third leading cause of cancer-related mortality worldwide, exhibiting substantial molecular and phenotypic heterogeneity. While surgical resection remains the treatment of choice for early-stage gastric cancer, the prognosis for advanced cases is considerably worse, with median survival rarely exceeding one year in cases where surgery is not feasible [1]. Notably, about two-thirds of gastric cancer cases are diagnosed at an advanced stage, complicating treatment efforts [2]. Management of advanced gastric cancer typically involves multimodal strategies, such as combining preoperative chemoradiation therapy, molecularly targeted therapy, and immunotherapy. Standard first-line chemotherapy includes a combination of platinum-based drugs and fluoropyrimidine, whereas second-line treatments often involve agents such as irinotecan and paclitaxel. Three-dimensional conformal radiation therapy is frequently employed to maximize the targeting of tumor tissues while minimizing damage to surrounding healthy tissues. Several therapeutic agents have received FDA approval specifically for treating gastric cancer, including trastuzumab for HER2-positive tumors; the antiangiogenic agent ramucirumab; and immune checkpoint inhibitors such as nivolumab, pembrolizumab, and sintilimab [1,[3], [4], [5]]. Despite these therapeutic advances, outcomes for patients with advanced gastric cancer remain poor. A comprehensive retrospective analysis highlighted the significant variability in median survival, with patients receiving surgery and chemotherapy showing a median survival of about 14.2 months, whereas those who did not undergo surgery only 7.0 months [6]. This stark contrast in survival outcomes underscores the need for deeper insights into the molecular mechanisms driving gastric cancer progression, therapeutic resistance, and recurrence [7]. With ongoing advancements in cancer research, there has been an increasing focus on cellular signaling networks. In particular, the STAT3 pathway and its associated signaling cascades have garnered substantial attention because of their critical roles in tumor development and therapeutic resistance. Further exploration of these pathways could lead to more effective treatment strategies for advanced gastric cancer, thereby reducing recurrence and extending patient survival (see Table 1, Fig. 1, Fig. 2, Fig. 3, Fig. 4, Fig. 5, Fig. 6, Fig. 7).

## 2 The basic structure and physiological function of STAT3

Signal transducer and activator of transcription 3 (STAT3; 770 amino acids) is encoded by a gene located on chromosome 17 [8]. STAT3 belongs to the highly versatile STAT family, which comprises STAT1 through STAT6. As a multifaceted transcription factor, STAT3 plays critical roles in various cellular processes, including cell proliferation, differentiation, survival, inflammation, immune regulation, and vasculogenesis [9]. It serves as a key mediator between extracellular signals and nuclear transcriptional responses. Structurally, STAT3 contains six distinct yet interdependent functional domains: the N-terminal domain (NH2), coiled-coil domain (CCD), DNA-binding domain (DBD), linker domain, Src homology 2 (SH2) domain, and transactivation domain (TAD) located at the C-terminus [10,11]. Under quiescent conditions, STAT3 remains latent and is tightly regulated by various inhibitory mechanisms. These include protein inhibitors of activated STATs (PIAS), suppressor of cytokine signaling (SOCS) proteins, tyrosine phosphatases such as SHP1, SHP2, and members of the PTP family, and ubiquitin ligases [12]. STAT3 activation is typically initiated by the binding of cytokines and growth factors, leading to phosphorylation mediated by receptor-associated JAK kinases. This phosphorylation triggers STAT3 dimerization and its subsequent translocation to the nucleus, where it regulates gene transcription [[13], [14], [15]].

STAT3 interacts with multiple ligands, including interferons, epidermal growth factor (EGF), and interleukins such as IL-5 and IL-6 [16]. Upon transient phosphorylation, STAT3 transduces signals from membrane-bound cytokine receptors to the nucleus, ultimately influencing gene expression [16]. STAT3 is essential for normal ontogenesis, particularly in maintaining the self-renewal of embryonic stem cells (ESCs). In mouse models, STAT3 deficiency leads to early embryonic lethality, underscoring its importance in development [17,18]. Moreover, STAT3 plays significant roles in immune regulation, enhancing the proliferation of neutrophils and B lymphocytes, facilitating monocyte differentiation, and promoting thymic T-cell survival [17]. Recent studies have also suggested that STAT3 may be involved in DNA replication, as it was found to interact with the WDHD1 promoter [19]. The role of STAT3 in oncogenesis and cancer progression has garnered substantial attention. STAT3 is a key regulator of cancer-related inflammation and is frequently activated in malignant cells. It regulates the expression of numerous oncogenes while concurrently inhibiting antitumor immune responses, contributing to poor clinical outcomes. In cancer, STAT3 is involved in a complex network of signaling pathways (including NF-κB and IL-6) and alternative splicing events, resulting in the production of two isoforms: STAT3α and STAT3β [10,16,20,21]. Unlike many other oncogenic proteins, somatic mutations in STAT3 are rare; however, STAT3 interacts with several oncogenes, including c-myc, c-Jun, and various kinases and growth factors, to drive tumorigenesis [20]. The STAT3 signaling pathway plays a pivotal role in the pathogenesis of gastric cancer, promoting tumor proliferation, resistance to therapy, and metastatic progression. Preclinical studies have demonstrated the efficacy of STAT3 inhibitors in controlling gastric cancer, highlighting the potential of targeting this pathway for therapeutic intervention. This article provides an in-depth analysis of the role of STAT3 in gastric cancer, offering a comprehensive foundation for future research aimed at developing novel and effective treatment strategies. The molecular network related to the basic structure and physiological function of STAT3 outlined in this chapter is shown in Fig. 1.

## 3 STAT3 and the induction of gastric cancer

Several studies have shown that the sustained activation of STAT3 can play a carcinogenic role within the gastric ecosystem. The IL-11/STAT3 axis is crucial for tumorigenesis within the gastric environment, as demonstrated in the gp130757FF mouse model, with similar results found in several murine models of gastric cancer [22]. Gastric cancer manifests as two primary histological variants, namely, the intestinal type and the diffuse type [23]. The intestinal type necessitates progression through a precursor condition known as intestinal metaplasia (IM). Duochen Jin reported that high levels of deoxycholic acid (DCA) (a key bile acid (BA) in duodenogastric reflux) are associated with STAT3 activation during the progression from chronic gastritis to IM. A combination of 16S rRNA gene sequencing and LC-MS analysis of gastric cancer tissue samples revealed that DCA activated the TGR5/STAT3/KLF5 pathway in epithelial cells, thus promoting cellular proliferation, hindering apoptosis, and stimulating the expression of proinflammatory cytokines and IM biomarkers. This cascade also includes STAT3 phosphorylation, its nuclear translocation, and interaction with the KLF5 promoter. These changes highlight a pathway through which gastric cellular changes facilitate the development of intestinal-type gastric carcinoma [24]. Similarly, Wang et al. reported that taurodeoxycholic acid (TDCA) from bile acids can act as an activator of the IL-6/JAK1/STAT3 pathway, promoting abnormal proliferation in normal gastric epithelial cells (GES-1) [25]. Overall, these findings indicate that bile reflux and STAT3 activation play pivotal roles in the emergence of gastric cancer.

Jian-Guo Zhang and colleagues used the PV9000 method for immunohistochemistry to examine Cripto-1 (CR-1) and phosphorylate STAT3 (p-STAT3) in various gastric samples. The samples assessed included 178 cases of gastric cancer (GC), 95 instances of matched normal gastric tissues, and lesions classified under chronic atrophic gastritis (CAG, 40 cases), intestinal metaplasia (IM, 48 cases), and dysplasia (DYS, 25 cases). Their findings revealed a significant increase in CR-1 and p-STAT3 levels within CAG (65.0 % & 60.0 %), IM (83.3 % & 77.1 %), DYS (80.0 % & 68 %), and GC (71.3 %) compared with their respective levels in normal gastric samples (43.2 % & 41.1 %, respectively; P < 0.05). Additionally, compared with nonmetastatic tissues, GC tissues that spread in lymph nodes presented high levels of CR-1 and p-STAT3 expression (78.3 % and 66.7 %, respectively) (53.1 % and 42.9 %, P < 0.05). The expression of CR-1 was significantly associated with histological changes and Lauren's classification of GC (P < 0.001). Additionally, the expression of CR-1 was found to be positively correlated with p-STAT3 expression in GC, which indicated that CR-1 plays pivotal roles in the emergence and lymphatic spread of gastric cancer (r(k) = 0.189, P = 0.002) [26]. The involvement of gastric cancer stem cells (GCSCs) may underlie these phenomena. These cells, derived from a subset of multipotent gastric stem cells (GSCs) residing within the gastric mucosa, undergo specific transformation, dedifferentiation, and excessive proliferation [27]. Cripto-1 and STAT3 are implicated in the formation and expansion of the gastric cancer stem cell population [28,29], with concurrent increases in p63 protein activity noted [129]. Additionally, a separate study reported elevated p63 levels in about 4 % of 355 gastric cancer patients, which are often associated with lymph node metastasis [30]. Currently, for individuals at high risk for gastric cancer, such as those with a familial genetic predisposition, early diagnosis typically involves endoscopic examination of the gastric mucosa, with suspicious samples being extracted for laboratory biopsy [31]. In the future, the widespread adoption of advanced techniques, such as flow cytometry, immunofluorescence labeling, and single-cell sequencing, could enable the detection of gastric cancer stem cells and their associated markers (e.g., Cripto-1, STAT3, and p63) in biopsy samples obtained via endoscopy. This approach has the potential to increase the accuracy of predicting gastric cancer risk in high-risk populations with significant genetic susceptibility or other risk factors, thereby facilitating more effective early screening and intervention. Hazel Tye reported that in mouse models, STAT3 directly promoted the expression of TLR2 (an inflammatory mediator found on the surface of certain cells) in gastric tumors, thus facilitating progression from chronic gastric inflammation to carcinoma. Clinical observations have shown that increases in STAT3 pathway activity and TLR2 expression are associated with decreased survival in GC patients [32]. XiaoYan Wang and colleagues conducted experiments on Wistar rats and reported that, after STAT3 is activated through tyrosine signaling, it significantly advances gastritis toward gastric carcinogenesis by modulating the expression of vascular endothelial growth factor (VEGF). These findings revealed that STAT3 plays a key role in the development of gastric cancer. Xiaoqin Wu reported that interleukin-17 (IL-17) can cause upstream activation of this pathway and increase microvascular density in gastric cancer tissues. On the basis of these findings, they proposed a composite clinical approach involving IL-17 inhibitors, STAT3 inhibitors, and VEGF-targeted medication (e.g., bevacizumab) to achieve more favorable therapeutic outcomes [33,34]. Hongyan Qi and colleagues determined that STAT3 signaling plays a key role in initiating gastric cancer, hyperactivating MSK1 kinase, which phosphorylates histone H3 serine 10 (H3S10) and STAT3, thus establishing a positive feedback loop. Further investigation revealed that NFATc2 is a new target within the STAT3-MSK1 regulatory spectrum. The interaction between STAT3 and MSK1 at the NFATc2 promoter drives H3S10 phosphorylation-dependent transcription, which in turn activates inflammatory pathways associated with gastric cancer genesis. Inhibiting the STAT3/MSK1/NFATc2 axis, including calcium blockers, can substantially decrease the proliferation and growth of gastric cancer cells in xenograft models [35]. Trefoil factor 1 (TFF1) is a secretory protein that plays a pivotal role in maintaining the integrity and barrier function of the gastric mucosa. In mice with TFF1 knockout (KO), the absence of TFF1 expression leads to an inflammatory phenotype and triggers a cascade of gastric lesions, including low-grade dysplasia, high-grade dysplasia, and adenocarcinoma. Research conducted by Wael El-Rifai's team revealed that TFF1 is frequently lost in the proteome of human gastric cancer cells. Restoration of TFF1 eliminates nuclear p-STAT3Y705 expression, inhibits interleukin-6 (IL6)-induced STAT3 transcriptional activity, and consequently suppresses the expression of its downstream target genes, such as Vegf, c-Myc, Birc5, and Il17a. These findings suggest that TFF1 can suppress gastric tumorigenesis by inhibiting the IL6-STAT3 proinflammatory signaling axis [36].

Zhichen Pu and colleagues reported that high levels of the circular RNA circCUL3 are significantly associated with advanced clinical stages and a decrease in the survival of individuals with gastric cancer (GC). They reported that circCUL3 enhances several cellular processes in GC cells, including cell proliferation, glucose utilization, lactate generation, ATP production, and the extracellular acidification rate (ECAR). They showed that circCUL3 increases the expression of hexokinase 2 (HK2) by capturing miR-515-5p and subsequently enhances STAT3 activity. These results suggest that the circCUL3/miR-515-5p/STAT3/HK2 pathway is a critical regulator of the Warburg effect (a metabolic phenomenon in cancer cells). Their study provided insights into pathways involved in the development of GC [37].

*Helicobacter pylori* and the inflammation caused by this bacterial species have been implicated in the pathogenesis of gastric cancer on the basis of molecular studies [38]. Chronic infection by the CagA strain of *H. pylori* can activate STAT3 in murine and human models, which in turn activates a cellular cascade through which STAT3 regulates the Th-17 lineage and its downstream IL-17 effector functions through IL-23 signaling pathways regulated by *H. pylori*. These changes make conditions favorable for the neoplastic transformation of the gastric epithelium [22]. Xing Zhang and colleagues determined the molecular relationships among *H. pylori*, inflammatory responses, and FGFR4 in gastric cancer pathology. We found that *H. pylori* and its related inflammation can activate FGFR4. This initiation can stimulate the nonreceptor tyrosine kinase activity of the SRC oncogene, leading to the downstream activation of STAT3. These changes can promote pathways that favor DNA repair, hinder apoptosis, and facilitate the carcinogenesis of gastric cells [39]. Juan-Yu Piao and colleagues reported alternate phosphorylation of STAT3 at serine 727 (P-STAT3Ser727) in the mitochondria of human gastric AGS cells independently after *H. pylori* infection. This process complements the classical activation process. Infected AGS cells exhibit mitochondrial distress, including the loss of integrity and swelling, coupled with an increase in the expression of LC3, which is a marker that indicates the presence of autophagic vesicles. These changes suggest that mitochondrial dysfunction is related to the serine-specific phosphorylation of STAT3, which contributes to cancer progression [40].

Ting Li and colleagues investigated cellular feedback mechanisms and reported an IL6-induced positive regulatory circuit in which miR-520d-5p modulates cyclophilin B (CypB) mRNA, influencing the growth of gastric cancer via changes in STAT3 phosphorylation. The suppression of miR-520d-5p by IL6 was found to depend on STAT3; these changes increased considerably when proinflammatory cues were present due to *H. pylori* infection, which promoted gastric carcinogenesis [41]. Overall, STAT3 is a critical factor that influences anomalous cellular events in gastric cells, ranging from proliferation to metabolic equilibrium, immune responses, and microvascular modifications. These changes depend on environmental shifts in the gastric environment. These disturbances may result in the emergence and progression of precancerous alterations, leading to the early onset of gastric cancer. The molecular network related to STAT3 and gastric cancer induction outlined in this chapter is shown in Fig. 2.

## 4 STAT3 and the growth and progression of gastric cancer

The STAT3 protein plays a pivotal role in the proliferation and development of gastric cancer. It influences multiple domains, including noncoding RNAs, signal transduction pathways, and epigenetic modulatory pathways. Arvind Pandey and colleagues reported that high levels of survivin were present in gastric adenocarcinoma tissues, both in the cytoplasm (90.8 %) and the nucleus (87.7 %). In premalignant, dysplastic gastric lesions, significant but moderate levels of survivin were present in the cytoplasm (56.7 %). Correspondingly, increases in the levels of native and phosphorylated STAT3 were detected in gastric cancer tissue, suggesting that survivin and STAT3 are crucial for the progression of this disease. The potential of these proteins as biomarkers and therapeutic intervention points should be further assessed to elucidate the synergistic effects of survivin and STAT3 on the progression of gastric cancer [42]. Considering that survivin, encoded by the BIRC5 gene, is entirely absent in normally differentiated terminal cells [43] and that the transcription initiation site of this gene is believed to be a binding site for the Sp1 complex—a cluster comprising two overlapping putative Sp1 binding sites [44]—it is noteworthy that previous studies have reported the cooperative induction of RhoU small GTPase and a set of atypical WNT response genes by SP1 and STAT3 in breast cancer models, contributing to poor prognosis [45]. We can reasonably infer that the overamplified and activated STAT3 factor in gastric cancer can directly or indirectly interact with the Sp1 complex binding sites of the BIRC5 gene, thereby activating the otherwise silenced BIRC5 gene and promoting the expression of survivin, which accelerates the proliferation of gastric cancer cells. Antagonizing STAT3 and blocking Sp1 binding sites could inhibit the proliferation of gastric cancer cells.

Tingting Xu investigated the role of miR-26b-5p and reported that its expression was lower in gastric cancer (GC) cells and tissues than in normal gastric epithelium and adjacent noncancerous gastric tissue. The lower levels of miR-26b-5p were related to accelerated proliferation of GC cells and adverse patient prognoses. By targeting phosphodiesterase 4B (PDE4B) and cyclin-dependent kinase 8 (CDK8), this miRNA modulates activity by reducing its phosphorylation and nuclear migration, impeding the progression of GC. The transcription of miR-26b-5p is suppressed by STAT3; thus, a feedback mechanism is established that increases the proliferation of gastric cancer cells when the level of miR-26b-5p is low or when STAT3 is overactivated [46].

Further investigation of noncoding RNA dynamics revealed that the overexpression of circABCA5 in GC facilitated SPI1 protein interaction and promoted the nuclear relocation of SPI1. The activation of the IL6/JAK2/STAT3 signaling cascade by SPI1 promotes gastric cancer malignancy and proliferation [47]. Mingdian Lu investigated LINC00467 and miR-27b-3p and reported that a decrease in LINC00467 levels increases the level of miR-27b-3p, which in turn inhibits STAT3 and reduces the growth of GC cells. Thus, miR-27b-3p acts as an inhibitor of STAT3, similar to the effect of miR-26b-5p, thus encouraging further inquiry into similar feedback mechanisms [48].

MicroRNA-21 (miR-21) is a small noncoding RNA that is expressed at high levels not only in gastric cancer but also in various solid tumors. Janson reported that in gastric adenocarcinoma mouse models, STAT3 signaling increased the expression of miR-21, which revealed a link between the STAT3 and miR-21 levels [49]. Wu's team discovered that a microRNA, miR-18a, a member of the miR-17–92 cluster, can positively regulate the activity of STAT3 by partially negatively regulating the STAT3 inhibitory gene PIAS3, thereby promoting the progression and spread of gastric adenocarcinoma [50]. In addition to PIAS3, genes such as SOCS and PTP can also inhibit STAT3. Increasing the activity of these genes holds promise for effectively slowing the progression of various cancers, including gastric cancer. However, the roles of these genes in tumorigenesis and antitumor treatment, such as the inhibition of the tumor suppressor gene p21, are controversial and require further investigation [51]. Research has long established that inactivation of the tumor suppressor gene PTEN occurs frequently in gastric cancer, affecting about 20 % of all cases [52]. Further investigation by Jian Wang revealed that in PTEN-deficient cancer cells, a deficiency in PTEN is positively correlated with low STAT3 activity, likely due to hyperactivation of the PI3K/AKT/mTOR pathway. Elevated STAT3 activity induces resistance in PTEN-deficient cancer cells to PI3K/AKT/mTOR inhibitors. However, the combined use of STAT3 inhibitors with PI3K/AKT/mTOR inhibitors can synergistically induce apoptosis in PTEN-deficient cancer cell lines, highlighting the potential of this combined therapeutic strategy in the treatment of PTEN-deficient tumors, including gastric cancer [53]. In contrast, research by Bao Gui Zhang suggested that microRNA-21 promotes tumorigenesis and invasion in gastric cancer by targeting the PTEN gene [54]. This forms a closed molecular loop. The enzyme EZH2 acts as a catalyst of polycomb repressive complex 2 (PRC2) and methylates lysine 27 of histone 3, which is a key step in the modulation of gene expression during cell differentiation. It acts synergistically with STAT3, where EZH2 catalyzes the phosphorylation of a tyrosine residue of STAT3, thus increasing its transcriptional activity. This reciprocal reinforcement aggravates the severity of TNM classification and the poor survival of patients. This finding implies that the EZH2-STAT3 system is an important target for developing effective strategies for treating gastric malignancies [10,55,56].

Similarly, the transcription of the long noncoding RNA (lncRNA) NEAT1 increases considerably in gastric cancer cells; this increase is similar to the increase in the expression of STAT3 and is correlated with an increase in neoplastic proliferation and invasion capacity. NEAT1 increases the transcription of STAT3 by suppressing miR-506, an inhibitor of STAT3 [57]. Z M Bai and colleagues reported a prominent decrease in hsa-miR-328-3p levels in gastric adenocarcinoma tissues, along with an increase in the levels of STAT3 and its downstream effectors, including MMP2, CCND1, and COX2. The administration of propofol arrested the proliferation of the SGC-7901 gastric cancer cell line, which was similar to the results recorded after the upregulation of hsa-miR-328-3p and the reduction in the levels of STAT3 and its downstream proliferative genes. This inhibitory effect of propofol, however, decreased following the knockdown of hsa-miR-328-3p, which showed that it can block the proliferation of gastric adenocarcinoma by reducing the intensity of the STAT3 signal [58]. Yi-Ping Liu reported that propofol can block the STAT3 pathway and inhibit the proliferation of gastric cancer cells by increasing the expression of miR-125b-5p [59]. This interaction also highlights the complex molecular interplay governing the progression of gastric cancer.

To determine the clinical outcomes of patients with gastric cancer (GC), the long noncoding RNA LOC339059, which is associated with the regulation of PD-L1 and its influence on macrophage polarization via the IL-6/STAT3 pathway, can be used as a prognostic marker. An evaluation of cellular interactions revealed that the association of LOC339059 with c-Myc leads to a decrease in the affinity of c-Myc for the IL-6 promoter. This reduction in interaction decreases the transcriptional activation of IL-6, consequently attenuating the IL-6/STAT3 signaling-mediated expression of PD-L1 and M2 macrophage polarization, thus slowing the progression of gastric cancer [60].

In a study on gastric cancer, the alkaline threonine zipper transcription factor ATF-like 3 (BATF3) was found to act as a key player in cell proliferation. Zhangyu Li reported that BATF3 levels were increased in gastric cancer specimens. Targeted silencing of BATF3 can decrease cell growth and resistance to radiotherapy, which is mediated by the upregulation of S1PR1 and increased phosphorylation of STAT3, while simultaneously facilitating radiation-triggered apoptosis. These findings indicate that BATF3 can act as a facilitator of STAT3 activity and thus increase the proliferative and radioresistance capacities of gastric cancer cells [61]. Similarly, the concentration of the circular RNA FCHO2 (circFCHO2) was found to be increased in gastric cancer (GC) patients and was associated with disease escalation and unfavorable outcomes. Zhe Zhang showed that circFCHO2 might trigger the JAK1/STAT3 axis by repressing miR-194-5p. These findings revealed a new way by which STAT3 levels might be altered in gastric cancer cells [62].

Serine hydroxymethyltransferase 2 (SHMT2) plays a key role in one-carbon metabolism and influences the proliferation and growth of cancer cells. Weida Wang reported that SHMT2 levels are increased in gastric cancer tissues and increase the stability of hypoxia-inducible factor 1α (HIF1α). In gastric cancer cells, SHMT2 modulates the stability of HIF1α, thus controlling a network of hypoxia-responsive genes that have regulatory effects on complementary pathways, including VEGF and STAT3. In vivo experiments have shown that knocking down SHMT2 can considerably decrease the expansion of GC tumors, suggesting a therapeutic effect focused on the inhibition of SHMT2 and HIF1α, which in turn can prevent the activation of the STAT3 pathway [63]. By investigating circular RNAs in gastric malignancies, Chenghui Li and colleagues reported that the circular RNA circBGN can directly interact with miR-149-5p. By preventing miR-149-5p from interacting with the IL6 mRNA, circBGN lifts the miRNA-imposed repression of IL-6 transcription, thus activating the IL6/STAT3 signaling pathway. This activation promotes proliferation and increases the severity of gastric cancer [64], highlighting the multifaceted regulatory mechanisms driving gastric cancer progression and revealing promising targets for intervention.

The intricate network of signals within the tumor microenvironment is closely associated with the emergence and progression of cancer. Bo Wei and colleagues reported that CD44 (a membrane protein) is expressed at variable levels in metastatic gastric cells and is closely associated with the proliferation and aggressiveness of these cancer cells, while it is inversely related to the level of miR-373. The hormone isoproterenol, which is associated with stress responses, was found to upregulate CD44 expression. Isoproterenol stimulates the activation of STAT3 via the β2-adrenergic receptor (β2-AR). After activation, STAT3 impedes the binding of miR-373 to its promoter and considerably increases the expression of CD44. This change in expression reveals the critical role played by the β2-AR/STAT3/miR-373 pathway in altering the phenotypic changes in gastric cancer cells, highlighting the role of tailored interventions in affected individuals [65]. Clinically, a highly positive association occurs between the IL6-STAT3 pathway and the activation of the checkpoint proteins PD-1 and PD-L1 on the surface of gastric cancer cells. Retrospective clinical data revealed that high levels of IL-6, p-Stat3, and PD-L1 are associated with a decrease in the integrity of the gastric cancer environment, which is correlated with suboptimal patient survival rates. The triad of “IL-6+p-Stat3+PD-1+ cell differentiation” is a key indicator of survival and is more reliable than the traditional TNM classification in determining postsurgical gastric cancer prognosis [66]. Xiang-Lei Yan and colleagues reported that miR-375 levels decrease considerably in gastrointestinal malignancies. Using mouse models, they showed that high miR-375 levels can decrease the activity of the JAK2/STAT3 pathway, leading to the downregulation of PD-L1 in gastric cancer. These findings indicate that miR-375 can play a key role in therapeutic interventions [67]. In a related study, Jing Zhao and colleagues reported that the lncRNA PVT1, which is overexpressed in gastric cancer tissues, is a marker for dense microvasculature and poor prognosis. PVT1 can interact with phosphorylated STAT3 in the nucleus and increase its stability. Thus, it can stimulate a cascade of events culminating in the reinforcement of the STAT3 pathway and an increase in VEGFA levels, which in turn can promote angiogenesis. Consequently, high levels of PVT1 and VEGFA often indicate poor survival [68].

The retromer complex subunit VPS35, which plays a signaling cargo retrieval role, is associated with neurodegenerative disorders and cancer progression. Qingqing Zhou and colleagues highlighted the role of VPS35 in increasing the growth and metastatic spread of gastric cancer cells through the IL-6/STAT3 signaling pathway. They reported that STAT3 initiates the transcription of VPS35 by interacting with its promoter and activating a self-perpetuating regulatory feedback circuit [69]. Xenia L and colleagues investigated the role of DC-specific intercellular adhesion molecule-3-binding nonintegrin-related protein (DC-SIGNR) in gastric oncology and reported that DC-SIGN attenuation decreases the activity of the JAK2/STAT3 axis, whereas DC-SIGN overexpression triggers activation of the pathway. By assessing the lncPath microarray via qRT-PCR assays, researchers reported that the abundance of the lncRNA RP11-181G12.2—an RNA—increases after the repression of DC-SIGN. Silencing this lncRNA increased the levels of DC-SIGN in gastric cancer cells and increased the activity of the JAK2/STAT3 signaling cascade [70]. Shuangming Lin and colleagues investigated miR-3184-5p and reported that it can repress various signaling pathways, including p-STAT3. It can also prevent BCL-2-mediated autophagy in gastric cancer cells. A decrease in the expression of miR-3184-5p in patients with gastric malignancies can increase the survival of cells by manipulating key oncogenic pathways, such as the AKT, STAT3, and IRE1 pathways. Thus, the expression of miR-3184-5p can act as an auxiliary biomarker that may reflect the clinical severity of gastric cancer [71].

Chao He and colleagues investigated the link between the lncRNA RPSAP52 and different types of cancer and reported that its depletion prevents cell proliferation and triggers cell cycle arrest at the G0-G1 checkpoint in gastric cancer (GC) cells while accelerating their apoptosis. They reported that miR-665 is an important target affected by the lncRNA RPSAP52 and elucidated the ceRNA inhibitory dynamics at play between them. They also reported that miR-665 inhibits the functions of STAT3. An increase in the expression of miR-665 and a deficiency in STAT3 reflect these antiproliferative effects on GC cell populations. Hence, the effect of the lncRNA RPSAP52 on the miR-665/STAT3 axis can promote the proliferation of GC cells [72].

The pri-miR-124-1 rs531564 polymorphism located in the peripheral blood DNA of GC patients has been associated with variations in STAT3, a correlation that might have significant pathogenic effects and provide insights into novel investigative leads [73]. In terms of local inflammatory effects, the levels of C3 and its derivative C3a are considerably greater in GC tissues than in noncancerous tissues, which closely matches the TNM staging criteria. Yuan and colleagues reported that introducing extraneous C3 increases the activity of p-JAK2/p-STAT3 in various GC cell lines. However, inhibiting C3 can significantly decrease this activity, suggesting that C3 deposition can promote the progression of GC through aberrant activation of the JAK2/STAT3 pathway [74].

The continuously active Wnt/β-catenin signaling pathway is a critical contributor to the progression of GC. Chenchen Liu and colleagues reported that CCAT5 (a lncRNA under the regulatory effect of Wnt) is significantly upregulated in GC. The β-catenin/TCF3 complex activates the transcription of CCAT5 and prolongs unfavorable patient outcomes. CCAT5 can increase the growth of GC and increase its metastatic potential. By docking with the C-terminal segment of STAT3, CCAT5 counters SHP-1-mediated dephosphorylation at tyrosine 705, thus promoting the nuclear translocation and transactivation of STAT3 and facilitating the progression of GC. Wnt3a and β-catenin also activate the STAT3 pathway, and the interaction between CCAT5 and STAT3 influences the effect of Wnt on STAT3 activity and subsequent neoplastic progression [75]. These findings indicate that STAT3 signaling, along with an array of microRNAs and signaling molecules, strongly influences the growth and progression of GC. Further insights into these molecular pathways and inhibitory RNA/protein countermeasures can present compelling investigative avenues. This might include the application of molecular and genetic engineering, such as RNAi technology, to suppress RNAs and small molecules that can activate STAT3 expression. Thus, these research strategies can reveal key ways to retard the proliferation and progression of GC. The molecular network related to STAT3 and gastric cancer growth and progression outlined in this chapter is shown in Fig. 3, Fig. 4.

## 5 STAT3 and drug resistance in gastric cancer

The extensive use of chemotherapy, precision-targeted therapy, and immunotherapy for treating gastric cancer has increased the number of cases of drug resistance. This resistance is a critical factor that not only decreases the survival of patients but also decreases the quality of life of those with advanced stages of GC. The complexity of drug resistance in gastric cancer is mediated by several key elements, including the cell death mechanism, changes in the tumor microenvironment, the roles of noncoding RNAs, epigenetic modifications, and the processes underlying the epithelial-mesenchymal transition [3]. In this context, STAT3 acts as a key mediator, increasing the resistance of gastric cancer cells by interacting with several pathways and mechanisms, which are discussed in this article.

## 6 STAT3 and chemotherapy resistance in gastric cancer

Peng Deng and colleagues investigated the role of circVAPA in gastric cancer (GC) and its interaction with cisplatin (DDP) resistance. Clinical data revealed an increase in the expression of circVAPA in GC tissues compared with noncancerous tissues. A targeted reduction in circVAPA was associated with a decrease in the ability of GC cells to proliferate, migrate, and invade, along with an increase in the rate of cell apoptosis. Moreover, in the SGC7901/DDP cell line, which is resistant to DDP, the expression of circVAPA, STAT3, and its responsive genes was found to increase. With respect to its mechanism of action, circVAPA captures miR-125b-5p, a microRNA that targets STAT3, thus increasing the ability of STAT3 to increase resistance to DDP [76]. Similarly, Laurino et al. reported that activation of the IL6/STAT3 axis can reduce the responsiveness of gastric cancer to various chemotherapeutic drugs, including cisplatin [77].

Xue Xiang and colleagues investigated the mechanism underlying chemotherapy-induced resistance in gastric cancer tissues and cancer stem cells. They reported that after treating gastric cancer cells with 5-fluorouracil (5-Fu) and DDP, the level of the miRNA miR-877-3p increased, and the level of the suppressive protein SOCS2 decreased, which suggested that miR-877-3p can regulate the oncogenic functions of gastric cancer cells by suppressing the target gene SOCS2. Additionally, after treatment with miR-877-3p, the Jak2/Stat3 signaling pathway, a downstream target of SOCS2, was found to be activated in vitro and in vivo. Similar effects were also found after treatment with the colony-stimulating factor GM-CSF, suggesting a new mechanism of chemotherapy-induced gastric cancer stem cell differentiation and resistance via GM-CSF-miRNA-Jak2/Stat3 signaling. These findings provide experimental evidence supporting the reduction of adjuvant chemotherapy doses in gastric cancer treatment [78].

Lijuan Ma reported that the JAK2/STAT3 signaling axis plays a key role in mediating endoplasmic reticulum stress, thus influencing the response of gastric cancer cells to 5-FU therapy and their autophagic processes, with ATF6 at the operational core. These findings suggested that inhibiting the JAK2/STAT3 pathway can decrease resistance to 5-FU in gastric cancer, which in turn can increase the efficacy of 5-FU treatment [79]. Shumin Ouyang and colleagues discovered a novel mechanism by which STAT3 interacts with DNA elements in the promoter regions of FNR-associated genes, such as GPX4, SLC7A11, and FTH1, and influences their transcription. This interaction revealed a regulatory axis that increases the resistance of gastric cancer cells to 5-FU. Blocking STAT3 can activate ferroptosis in these cells by increasing lipid peroxidation and the accumulation of Fe^2+^; this is an effective strategy to counter 5-FU resistance in advanced gastric cancer patients [80]. Jun-Ling Zhang and colleagues investigated the human cervical cancer oncogene (HCCR) and reported its ubiquitous overexpression in various types of cancer; its levels were considerably high in gastric cancer cell lines, especially in 5-FU-resistant strains. They proposed a feedback loop in which STAT3 activation increases HCCR levels, and HCCR, in turn, increases p-STAT3 activity. This interaction greatly increases the invasiveness of gastric cancer cells and contributes to resistance to 5-FU treatment [81].

Jun Ma and In-Hye Ham reported that the number of cancer-associated fibroblasts is greater in drug-resistant gastric cancer tissues. These fibroblasts improve resistance to chemotherapy by secreting cytokines such as IL-11 and IL-6, which exert their influence predominantly through the anti-apoptotic pathways of IL-11/IL-11R/gp130/JAK/STAT3 and IL6/JAK/STAT3. Additionally, the combined application of chemotherapeutic agents with JAK inhibitors can disrupt this pattern of resistance and is associated with better survival outcomes in gastric cancer mouse models with tumor transplants [82,83].

Tuo Ruan and other researchers reported that DCP1a is expressed at significantly higher levels in tumor tissues (p < 0.05), which is associated with poor prognosis. The downregulation of DCP1a in cells was found to be associated with a decrease in the proliferation rate, migration ability, and resistance to chemotherapy. Researchers have reported that the expression of MRP-1 and the activation of the AKT and STAT3 pathways are involved in this regulation. However, the limitation of this study was that it could not determine the causal relationship between DCP1a and regulatory factors such as STAT3 [84].

Pei Jou Chua and colleagues investigated the interaction between YB-1 and the JAK/STAT signaling pathway in the context of chemotherapeutic resistance in gastric cancer. They reported that although the reciprocal regulation between the two factors was not strongly strong, simultaneous knockdown of YB-1 and STAT3 led to synergistic inhibition of cell invasion and drug resistance in NUGC3 cells. These findings suggest that the combined application of YB-1 antagonists and STAT3 inhibitors is a promising strategy for treating gastric cancer [85].

Yanwei Ye and colleagues investigated the role of the Arg388 variant of fibroblast growth factor receptor 4 (FGFR4) in gastric cancer (GC). Compared with FGFR4-Gly388 patients, FGFR4-Arg388 patients were more likely to have a higher cancer stage, poorer survival, and significantly higher levels of wave proteins (p = 0.025) and p-STAT3 (p = 0.009). These strains were also found to have increased resistance to the chemotherapeutic drug oxaliplatin. These findings indicate that the FGFR4-Arg388/STAT3/epithelial-mesenchymal transition (EMT) pathway imparts resistance to oxaliplatin, suggesting that targeting this pathway may enhance the chemotherapeutic effect of oxaliplatin [86]. Arginine ADP-ribosylation is an enzyme-catalyzed, potentially reversible posttranslational modification in which the ADP-ribose moiety from NAD+ is transferred to the guanidino group of arginine [87]. This interaction may underpin the structural basis for the FGFR4-Arg388 regulation of the overexpression of downstream molecules such as STAT3. In colorectal cancer models, which share similar pathological environments and treatment approaches with gastric cancer, FGFR4 silencing has been shown to induce poly ADP-ribose polymerase-1 (PARP) cleavage. This cleavage is further enhanced in the presence of chemotherapy [88]. Moreover, Molly R. Ryan et al. reported that another member of the FGFR family, FGFR1, whose V561M gating mutation can drive lung cancer cells to develop resistance to AZD4547 through STAT3 activation and EMT [89]. Thus, we infer that the connection between FGFR mutation and STAT3 overexpression and tumor cell resistance may be widely present in a variety of cancers, including gastric cancer. Considering the current exploratory attempts to use PARP inhibitors in gastric cancer treatment [90], the addition of a PARP inhibitor (such as olaparib) to oxaliplatin chemotherapy for FGFR4-Arg388 gastric cancer patients is worth investigating. It is expected to inhibit FGFR4-Arg388 and then inhibit STAT3, reducing the possibility of drug resistance. If direct inhibitors against STAT3 can be used at the same time, the effect may be better. A study indicated that 29.6 % of gastric cancer tumors exhibited heterozygous mutations of this specific allele type, whereas 4.2 % had homozygous mutations [91]. This combination may reduce resistance and improve therapeutic efficacy. The molecular network related to STAT3 and chemotherapy resistance in gastric cancer outlined in this chapter is shown in Fig. 5.

## 7 Relationship of STAT3 with resistance in gastric cancer-targeted therapy and immunotherapy

The development of specific inhibitors targeting various receptor tyrosine kinases (RTKs), including EGFR, MEK, FGFR, and HER2, is a promising cancer-targeted therapeutic strategy. Hyperactivation of STAT3 can effectively bypass the inhibitory effects of targeted therapeutic strategies against RTKs, increasing the resistance of cancer cells to RTK inhibitors. These findings highlight the advantages of combining RTK inhibitors with STAT3 inhibitors to improve therapeutic outcomes [9]. In gastric cancer, trastuzumab is used as a principal treatment option for HER2-positive patients [92]. The overexpression of STAT3 helps HER2-positive gastric cancer cells develop resistance to trastuzumab, often with KRAS gene mutations [9]. Guangchao Li, Zhengyan Yang, and their research groups confirmed that sustained administration of trastuzumab can cause the development of resistance in gastric cancer cell lines. Long-term treatment with trastuzumab considerably decreases the phosphorylation of Akt, subsequently increasing the levels of fibronectin (FN), EGF, and IL-6. Together, these changes can result in hyperactivation of STAT3, which in turn can increase the expression levels of FN, EGF, and IL-6, thus initiating a self-reinforcing cycle that perpetuates STAT3 signaling. This increase in STAT3 activity promotes the expression of MUC1 and MUC4, which mediate trastuzumab resistance by persistently activating HER2 and decreasing the affinity of trastuzumab for binding to HER2 [93,94].

Cells resistant to chemotherapy often have characteristics akin to epithelial-mesenchymal transition (EMT) and possess traits similar to those of cancer stem cells, which increase their invasion and metastatic capabilities both in vitro and in vivo. Mei Hua Jin and colleagues reported that the activation of the Src protein, through its interaction with phosphorylated focal adhesion kinase (FAK), interferes with various downstream molecules, including STAT3, increasing resistance to trastuzumab. The application of the Src inhibitor bosutinib was found to counter this resistance [95]. Moreover, this biological cascade is similar to an increase in Notch signaling, characterized by an increase in the levels of Notch ligands such as Jagged-1 and increased expression of the Notch-regulated genes Hey1 and Hey2. The inhibition of the Notch and STAT3 pathways may increase the therapeutic potency of trastuzumab against gastric cancer and prevent the development of treatment resistance [94]. Kai-Wen Hsu reported that the levels of expression of Notch1 and Jagged1 are strongly associated with the levels of phosphorylated STAT3 and Twist in gastric cancer tissues. They showed that the Notch1/STAT3/Twist signaling pathway is involved in the progression of GC. Targeted modulation of this signaling pathway might provide novel strategies for combined therapeutic interventions [96].

Ke Chen investigated Rab31 and its role in improving cisplatin resistance and augmenting metastasis. A decrease in the level of Rab31 increases apoptosis and decreases sensitivity to cisplatin; the opposite effect occurs when Rab31 is overexpressed. Treatment with cisplatin and increasing Rab31 levels increase the expression of Twist1. However, the absence of Twist1 increases the susceptibility of STAD cells to platinum drugs, and a high level of Rab31 does not reverse this increased sensitivity. The activation of Twist1 by Rab31 facilitated through STAT3 stimulation and MUC-1 suppression reveals a novel Rab31/STAT3/MUC-1/Twist1/EMT axis implicated in the drug resistance and metastatic potential of gastric cancer [97].

In addition to evaluating the effectiveness of trastuzumab, some clinical studies have assessed the efficacy of lapatinib in the treatment of HER2-positive gastric cancer. For example, HP Kim exposed a HER2-positive gastric cancer cell line (SNU216) to lapatinib for a prolonged period, which led to the development of the lapatinib-resistant strain SNU216 LR. These cells not only resisted lapatinib but also exhibited cross-resistance to several EGFR and HER2 inhibitors, including gefitinib, cetuximab, afatinib, and dacomitinib. In resilient cells, key signal transduction pathways, such as the STAT3, MET, HER3, Akt, and MAPK pathways, are activated even in the presence of lapatinib. These findings demonstrated the effectiveness of siRNAs directed against proteins associated with the EMT phenotype in the context of acquired resistance to HER2-targeted therapy in HER2-positive gastric cancer [98].

In specific cases of gastric cancer, the receptor tyrosine kinase Met is either activated or amplified, which led to the development of targeted Met inhibitors. However, Andrea Z Lai and her team reported that patients often develop resistance to these small-molecule inhibitors. They reported significant similarities in the gene sets modulated by Met activation and those influenced by STAT3, in which STAT3 plays a central role in propagating Met-induced proliferative signals. Thus, the coapplication of Met and STAT3 inhibitors can minimize resistance and enhance treatment efficacy in gastric cancer [99].

Na Chen reported that FGD5, a Rho family guanine nucleotide exchange factor, acts as a key modulator of endothelial cell angiogenesis and apoptosis. His findings revealed that FGD5 can interact with EGFR, resulting in a decrease in the ubiquitination of EGFR and sustained activation of downstream signaling molecules, including STAT3 and pSTAT3 [100]. In the early phases of gastric cancer, dopamine and the cAMP-regulated phosphoprotein Mr 32000 (DARPP-32) are frequently overexpressed. Shoumin Zhu and colleagues conducted a series of in vitro assays and 3D gastric organoid cultures using mouse models and human tissues to determine the effect of DARPP-32 on the activation of IGF1R and STAT3 signaling, which is crucial for the onset of gastric cancer. Additionally, the binding of IGF1R to DARPP-32 can increase the phosphorylation of IGF1R, which in turn can trigger the downstream SRC and STAT3 pathways. Considering that the level of DARPP-32 increases along with positive STAT3 nuclear immunostaining in gastric cancer tissues, the DARPP-32-IGF1R signaling axis might act as a critical regulator of STAT3 signaling and play an important role in gastric tumorigenesis [101]. DARPP-32 can enhance the interaction between EGFR and ERBB3, thereby activating the phosphatidylinositol 3-kinase (PI3K)-AKT signaling pathway and promoting resistance to gefitinib in gastric cancer cells [102]. Additionally, gastric cancer cells overexpressing STAT3 often overexpress EGFR on the cell membrane [103]. We hypothesize that STAT3 may facilitate the development of EGFR-positive gastric cancer and contribute to drug resistance through interactions with molecules such as DARPP-32 and FGD5, possibly involving other yet-to-discovered mechanisms. Exploratory trials have already been conducted using EGFR-targeted therapies in gastric cancer [104]. In the future, the combined application of anti-EGFR targeted therapies and STAT3 inhibitors could represent a promising investigational treatment strategy for patients with EGFR-positive gastric cancer.

The widespread activation of STAT3 in tumor-infiltrating immune cells weakens innate and adaptive immune responses and facilitates immune evasion. The most potent killers of gastric cancer cells in the human immune system are CD8^+^ T cells [105]. Excessive STAT3 activity can disrupt the production of proinflammatory mediators such as IFNγ, hinder antigen presentation, and attenuate the cytolytic function of effector cells. In adaptive immunity, high STAT3 activity may also decrease the accumulation of CD8^+^ T cells, thus decreasing their antitumor effects. This process can be promoted by inhibiting the expression of the receptor CXCR3 for CXCL10 on CD8(+) T cells, thereby preventing the effective accumulation of CD8(+) T cells at tumor sites [16,106]. Hence, adjunctive STAT3 inhibition can increase the efficacy of T cells and enhance their affinity and cytotoxic action against cancer cells in combination with conventional immunotherapeutic agents, such as pembrolizumab, nivolumab, and sintilimab, which are administered for gastric cancer treatment. Considering the preliminary success of CAR-T-cell therapy in treating gastric cancer [107], future strategies might include employing genetic engineering to knock out or silence the STAT3 gene in CAR-T cells before they are reinfused into patients with gastric cancer. This approach could enhance the accumulation and cytotoxic efficacy of these cells at the tumor site.

Junquan Song conducted bioinformatics analysis across 33 different types of cancer, including gastric cancer, and reported that receptor-interacting protein kinase 2 (RIPK2) plays a key role. Compared with healthy tissues, cancerous tissues have higher RIPK2 levels, which are correlated with the prognosis of several types of cancer. An increase in the expression of RIPK2 facilitates resistance to innate and adaptive immune therapeutic techniques via pathways such as the IL-6/JAK/STAT3 signaling pathway, the interferon-γ response, and the interferon-α response; thus, RIPK2 plays an important role in gastric cancer [108]. The molecular network related to STAT3 and targeted therapy and immunotherapy resistance in gastric cancer in this chapter is shown in Fig. 6.

## 8 STAT3 and gastric cancer metastasis

Metastasis is a serious threat, significantly decreasing survival and reducing the quality of life of individuals with gastric cancer. The metastasis of cancer cells to distant locations often signals advancement to terminal Stage IV. Under such conditions, the therapeutic aim shifts to palliation—extending life and easing discomfort. Consequently, arresting and managing the spread of gastric cancer is the primary aim of oncological research endeavors. In the subsequent sections, we discuss the involvement of STAT3 in gastric cancer metastasis; this information might serve as a resource for researchers.

Mengyuan Liu and colleagues used bioinformatics and reported that RBMS1 is a putative gene that facilitates GC metastasis. These results suggested that the interaction between the transcription factors MYC and RBMS1 promotes the migration and invasion of cells. This effect is mediated by alterations in histone modifications within the promoter region, transactivation of IL6, and a subsequent increase in the activity of the JAK2/STAT3 signaling pathway. Thus, the inhibition of RBMS1 is an effective strategy to prevent gastric cancer metastasis [109].

Macrophages are highly versatile immune cells that are extensively distributed throughout the adult mammalian body and play pivotal roles in regulating homeostasis, inflammation, and antitumor immunity in tissue-specific and environment-dependent manners. The activation of these cells bifurcates into two pathways: the classic M1 and the M2 alternative. In tumors, infiltrative macrophages, also known as tumor-associated macrophages (TAMs), often adopt an M2 phenotype induced by environmental factors. This state suppresses the cytotoxic functions of immune cells that are effective at destroying tumors, thereby weakening antitumor immunity. Consequently, this leads to an immunosuppressive tumor microenvironment, which is closely associated with poor prognosis in cancer patients, including the propensity for distant metastasis [110]. While the activation of M2 macrophages is primarily mediated by the STAT protein family member STAT6, the role of STAT3 is equally indispensable in this process [110,111]. Ganggang Mu and colleagues investigated the tumor-associated macrophage (TAM) environment and reported that calmodulin 2 (CALM2) plays a key role. They showed that CALM2 regulates the JAK2/STAT3/HIF-1/VEGFA axis and promotes the polarization of M2 macrophages. This process, in turn, facilitates immune evasion and stimulates lung metastasis and angiogenesis within the pathological framework of GC, suggesting that CALM2 enables detrimental processes [112].

Hongkui Yang and colleagues assessed the link between STAT3 activation and the occurrence of peritoneal metastasis in gastric cancer (GC) patients. GC tumor tissues presented increased expression of activated STAT3, with higher levels recorded in cases involving spread in the peritoneal region. In vitro studies confirmed these findings, indicating that the activation of STAT3 increases the ability of GC cells to migrate and secrete mesenchymal transition factors. Suppressing STAT3 severely compromised the prevalence of peritoneal metastasis of GC in an animal model, which indicated that the pathway is directly associated with this metastatic behavior [113].

Jie Yu and her team investigated FYN, a gene that is variably expressed in GC and can regulate the metastasis of different types of cancer. Silencing FYN substantially reduced the motility and invasiveness of GC cells and decreased the formation of metastatic lung nodules in vivo. These findings suggested a positive interaction between an increase in the levels of EMT markers and FYN, which indicated that FYN is involved in EMT dynamics. Genomic analyses revealed a correlation between STAT3 signaling and FYN. Disruption of STAT3 activity reversed the inductive effect of FYN on EMT and inhibited metastasis, which suggested that FYN can drive GC metastasis through a mechanism involving STAT3-mediated EMT [114].

The IL-6/STAT3 signaling pathway also plays a role in this context. Through the polarization of macrophages to the M2 phenotype, which is driven by mesenchymal cells, this pathway can facilitate the transition of GC cells to an EMT phenotype, encouraging metastasis [115]. Cancer-associated fibroblasts (CAFs) are a complex and heterogeneous group of mesenchymal cells with robust proliferative capacity and diverse origins, including mesenchymal transition. These cells extensively contribute to the malignant progression of gastric cancer (GC) [116]. Guofang Lu and colleagues investigated the effect of CAFs on the prometastatic tumor microenvironment and reported that SLIT2 plays an important role. SLIT2 is produced by CAFs and was found to support GC metastasis in two distinct GC cell lines, AGS and MKN45, by interacting with the roundabout guidance receptor 1 (ROBO1). Proteomic analysis revealed that ROBO1 interacts with the serine/threonine kinase NEK9; the kinase domain of NEK9 is necessary for interacting with the intracellular divide (ICD) of ROBO1. NEK9 can directly phosphorylate TRIM28, an associated transcriptional modulator, and the protein CTTN. TRIM28 acts as a transcription elongation catalyst and activates CTTN. Researchers detected coordinated activation of CTTN transcription by STAT3 and NF-κB p100 in AGS and MKN45 cells, which indicated the presence of a multifaceted network driven by CAF-derived SLIT2/ROBO1/NEK9/TRIM28/CTTN/STAT3, suggesting several ways to inhibit the metastasis of GC [117]. Xiongyan Wu and colleagues concluded that cancer-associated fibroblasts (CAFs) isolated from gastric cancer can activate the JAK2/STAT3 pathway in gastric cancer cells by secreting IL-6. This activation enhances the epithelial-mesenchymal transition (EMT) and peritoneal dissemination of gastric cancer cells. Additionally, the deployment of neutralizing antibodies to deplete IL-6 or the application of the specific inhibitor AG490 to impede the JAK/STAT3 pathway significantly mitigates these CAF-induced phenotypic traits in gastric cancer cells and curtails their metastatic propensity [118]. Jianzheng Wang and colleagues also reported that bone marrow fibroblasts (BMFs) can increase the expression levels of IL-6, TGF-β1, STAT3, and p-STAT3, thus promoting gastric cancer metastasis in a mouse model [119]. These studies collectively reveal the presence of pathways through which cancer-associated fibroblasts (CAFs) mediate gastric cancer metastasis via STAT3-related mechanisms. These findings suggest that STAT3 may play a crucial role in shaping the heterogeneity of the fibroblast population within gastric cancer tissues and inducing their metastatic behavior, representing a noteworthy research direction. Importantly, the cytokine TGF-β, which has been suggested in several papers to mediate EMT transformation to form CAFs in gastric cancer, can be positively regulated by STAT3 [120,121]. Therefore, it can be reasonably inferred that in future precision targeted therapy, a combination of TGF-β inhibitors, CAF inhibitors and STAT3 inhibitors can be used in specific patients with a high risk of distal metastasis through genetic testing to minimize the risk of distal metastasis.

Li Xu and colleagues reported that interferon-induced transmembrane proteins (IFITM) in gastric cancer can regulate the expression and secretion of interleukin 6, which in turn can increase the expression of IFITM through feedback, thus mediating the growth of gastric cancer and lung metastasis. Hence, this process might also involve the activation of the IL-6/STAT3 pathway; however, further studies are needed to confirm this speculation [122].

Chao Wang and other researchers reported that the transcription factor homeobox A11 can promote the peritoneal metastasis of gastric cancer cells. This mechanism involves altering the stemness of cancer cells and subsequently increasing their adhesion, migration, invasion, and anti-apoptotic abilities, primarily through a positive feedback loop involving Stat3, where HOXA11 increases the phosphorylation of Stat3 (Tyr705) and induces its accumulation in the nucleus [123].

Yangbing Jin and colleagues reported that the interaction of EGR1/TGF-β1/CD44s/STAT3 signaling between mesenchymal cells and gastric cancer cells induces EMT and the stem phenotype, leading to the peritoneal metastasis of gastric cancer cells. Inhibiting this pathway can prevent widespread peritoneal metastasis of gastric cancer cells, which can save the lives of many advanced gastric cancer patients [124].

Miaomiao Pei and colleagues reported that the lncRNA LINC00501 is frequently upregulated in GC cells and tissues and is associated with adverse clinicopathological characteristics in patients with GC. LINC00501 interacts directly with the chaperone protein HSP90B1, which prevents ubiquitination of the target protein STAT3, thus helping maintain the stability and activity of STAT3. In turn, STAT3 can directly bind to the LINC00501 promoter and activate the expression of LINC00501, thereby forming a positive feedback loop and accelerating the growth, invasion, and metastasis of gastric cancer cells [125].

Xiangqian Xu and other researchers reported that miR-125b could affect the subcellular distribution of STAT3. Mimicking or static treatment with miR-125b inhibits the invasion and peritoneal metastasis of gastric cancer cell lines. In contrast, IL-6 can reverse this inhibition, indicating that this miRNA can inhibit the distant metastasis of gastric cancer by suppressing the IL-6-STAT3 pathway [126]. The molecular network related to STAT3 and gastric cancer metastasis in this chapter is shown in Fig. 7.

## 9 STAT3 inhibitors in the treatment of gastric cancer

Several studies have shown that the STAT3 pathway plays a key role in facilitating the onset, progression, drug resistance, and metastatic spread of gastric cancer. Thus, the development of STAT3 inhibitors can help combat this malignancy. Ongoing studies are investigating small-molecule inhibitors, naturally derived compounds, peptide mimetics, and related derivatives [127].

Jin Sung Koh and colleagues reported that pantoprazole, known for its use in managing gastric acidity, can also modulate oncogenic pathways. This medication upregulated SHP-1 and suppressed the expression of phosphorylated STAT3 in a dose-responsive manner in AGS and MKN-28 gastric cancer cells. Additionally, in xenograft models, the intraperitoneal administration of pantoprazole considerably reduces tumor size and exacerbates distal metastasis [128]. Dehua Yu and colleagues performed molecular docking and virtual screening and reported that trilinolein from marine sources is an inhibitor that directly interacts with STAT3. It inhibits the phosphorylation of STAT3, downregulates the expression of STAT3-dependent genes such as c-Myc and Cyclin D1, and has anticancer activity with minimal reported toxicity [129]. Yan Zhong and colleagues characterized the small molecule WZ-2–033, which disrupts the activation and dimerization of STAT3, exerting its effects across cancer models, including gastric cancer. By disrupting dimer formation and DNA binding, WZ-2–033 can inhibit STAT3-dependent transcription, thus attenuating cell proliferation and invasion at locations where STAT3 is aberrantly active [130]. Peng He and colleagues reported that the tri-phenyl triazole derivative HP590 can act as a dual inhibitor of STAT3 phosphorylation at Tyr705 and Ser727. HP590 selectively targets STAT3, prevents traditional and alternative activation mechanisms, and stops their related biological functions; these changes inhibit gastric cancer growth in various experimental contexts [131]. The effect of propofol, a drug that can inhibit gastric cancer cell proliferation by noncoding RNAs [58,59], is complemented by Xiaoxia Jiang's findings. Jiang and colleagues reported that HJC0152 impedes STAT3 activation, prevents its downstream gene expression, and induces apoptosis in gastric cancer lines with hyperactivated STAT3 [132]. Mingzhu Li and colleagues reported that vortioxetine acts as a dual inhibitor of JAK2/SRC, preventing cell proliferation by inhibiting the JAK2/SRC-STAT3 pathway, suggesting that it has anti-gastric cancer effects [7]. Xiaoqin Wu reported that the phosphorylation inhibitor JSI-124 could block STAT3 activation, suppress the expression of VEGF, and thus inhibit angiogenesis and the progression of gastric cancer [34]. Jiye Wang reported that cryptotanshinone could enhance the anticancer effect of doxorubicin by inactivating STAT3 and downregulating STAT3-regulated genes. These effects suggest promising therapeutic efficacy of combined treatment [133]. Yanxia Huang examined the therapeutic role of oxymatrine and reported that it can inhibit the JAK/STAT pathway in gastric cancer cells, a process that is reversed by the overexpression of IL-21R [134]. In-Hye Ham and colleagues reported that curcumin can counteract CAF-induced chemoresistance in GC tissues and can function synergistically with 5-fluorouracil (5-FU) to combat gastric cancer [135]. Xin Gan and colleagues investigated C7-bridged monocarbonyl analogs of curcumin and identified one compound that exhibited antigastric cancer activity by partly inhibiting the AKT and STAT3 pathways; their findings encouraged the investigation of this compound as a chemotherapeutic agent [136]. Tyrosine kinase inhibitors are the most widely studied type of targeted therapeutic drug for gastric cancer. Hui Zhu et al. investigated the therapeutic effect of the triple tyrosine kinase inhibitor nintedanib on gastric cancer and reported that in gastric cancer cell lines with stable overexpression of STAT3 and in nude mice bearing tumors, nintedanib could inhibit tumor growth and induce the autophagic death of gastric cancer cells by inhibiting STAT3 phosphorylation and upregulating the autophagy-related protein Beclin1 [137]. Haobin Li et al. studied the design and synthesis of a class of STAT3 degraders on the basis of proteolysis-targeting chimeras (PROTACs). They first synthesized an analog of the STAT3 inhibitor S3I-201 as a ligand and synthesized a series of PROTACs using pomalidomide, a ligand of cereblon (CRBN)/cullin 4A E3 ligase. Among them, SDL-1 degrades the STAT3 protein in vitro and has good anti-proliferative activity against gastric cancer cells, inhibits the invasion and metastasis of MKN1 cells, and induces apoptosis and cell cycle arrest in MKN1 cells [138]. Haobin Li et al. used the STAT3 inhibitor S3I-201 (a potent nonselective alkylating agent [139]) as the starting point for drug development. By introducing a special fragment, the naphthoquinone unit, into a STAT3 inhibitor, a series of new STAT3 inhibitors, 9a-x, were synthesized. Most of these compounds have shown strong anti-proliferative effects on various gastric cancer cell lines. The representative compound 9n (SIL-14) can effectively inhibit the cloning and migration of MGC803 gastric cancer cells at low micromolar concentrations in vitro, block the cell cycle and induce the apoptosis of MGC803 cells. In addition, SIL-14 can also inhibit the phosphorylation of the STAT3 protein and significantly reduce the expression of total STAT3, suggesting that it may play an anticancer role by blocking the STAT3 signaling pathway [140]. Table 1 shows some of the STAT3 inhibitors mentioned in this chapter for their application in gastric cancer.

**Table 1:** The molecular formulas and effects of the STAT3 inhibitors mentioned in this article.

STAT3 inhibitor types	Chemical Structure	Mechanism
Pantoprazole		Upregulates SHP-1 and downregulates p-STAT3 expression in a dose-dependent manner, reduces gastric tumor volume, and inhibits distant metastasis.
Terphenyllin		Inhibits STAT3 phosphorylation and activation and reduces protein levels of STAT3-dependent target genes, including c-Myc and Cyclin D1.
WZ-2–033		Can disrupt HA-STAT3 and Flag-STAT3 dimerization in intact cells, inhibit STAT3-DNA binding activity, inhibit phosphorylation and nuclear accumulation of pY705-STAT3, thereby inhibiting STAT3-dependent transcriptional activity and the expression of STAT3 downstream genes.
HP590		Selectively targets STAT3 and significantly inhibits its classical and noncanonical activation pathways (p-Tyr705 and p-Ser727) and corresponding biological functions, thereby inhibiting the growth of gastric cancer in vitro and in vivo.
Propofol		Increasing the content of inhibitory noncoding RNA (hsa-miR-328-3p and miR-125b-5p) inhibits the STAT3 pathway and thereby inhibits gastric cancer proliferation.
HJC0152		Inhibits the expression of activated STAT3 and its downstream target genes (c-Myc and clyclinD1) in GC cells, induces the expression of STAT3 overactivated gastric cancer cell lines, simultaneously downregulates survivin and Mcl1, and upregulates cleaved poly(ADP-ribose) 6 polymerization enzyme, limiting the metastasis and invasion of gastric cancer cells.
Vortioxetine hydrobromide		JAK2/SRC dual inhibitor inhibits STAT3 dimerization, nuclear translocation activity, and JAK2- and SRC-dependent gastric cancer cell proliferation.
JSI-124		Block STAT3 activation and significantly reduce VEGF expression, thereby preventing angiogenesis and inhibiting gastric cancer progression.
Cryptotanshinone (CPT)		CPT enhances the anticancer activity of doxorubicin (DOXO) against gastric cancer cells by inactivating STAT3 and inhibiting STAT3-regulated anti-apoptotic gene expression.
Oxymatrine (OMT)		It can inhibit the JAK/STAT signaling pathway to inhibit gastric cancer cells, but its inhibitory effect can be counteracted by the overexpression of the interleukin 21 receptor (IL-21R).
Curcumin		It can eliminate the activation of the JAK/STAT3 signaling pathway mediated by cancer-associated fibroblasts (CAF) in GC tissues, thereby counteracting the CAF-induced resistance of GC cells to the chemotherapy drug 5-fluorouracil (5-FU) and exerting Synergistic anticancer effect with 5-FU.
C7 Bridged Monocarbonyl Curcumin Analog (MCA) Synthetic Compounds		Partially inhibited the AKT and STAT3 pathways in gastric cancer cells, showed anti-gastric cancer activity in vitro and in vivo, and demonstrated good in vivo safety, providing potential compound selection for the development of future chemotherapeutic drugs.

*Statement: The last figure is from reference 98, the structure of HP590 is from reference 87, and the structures of other molecules were obtained from Wikipedia, ResearchGate, Google Patents, and the TargetMol website.

Despite these successful preclinical studies, thorough peer-reviewed, clinical trials related to STAT3 inhibitors for gastric cancer treatment are lacking in the main databases, such as ClinicalTrials.gov. The main challenges include inhibitor solubility, cellular uptake, general safety, and comprehensive toxicity. The complex nature of the oncogenic signaling of STAT3, its multiple alternate routes, and the uninvestigated compensatory pathways hinder the translational application of these molecular compounds in clinical therapy. The difficulty in developing a drug may also be related to the fact that the molecule has relatively shallow surface pockets, which generally makes it challenging for chemical substances to bind effectively [141]. In addition, considering the widespread occurrence of mutations and amplifications in molecules similar to STAT3 (such as KRAS and P53) in cancers such as gastric cancer, which are difficult to target with drugs, we propose two theoretical models to explain this phenomenon. One model, termed the optimal Hub model, posits that molecules such as STAT3, KRAS, and P53 serve as optimal hubs in several signaling pathways because of their inherent structural advantages (such as relatively small molecular weights and resistance to inhibitor binding). They act as lightweight, cost-effective intermediaries in these pathways and are not indispensable components; when inhibited, these pathways can compensate via alternative routes, minimizing the overall impact on cancer cells and tumor growth. Conversely, certain crucial gene mutations (e.g., EGFR driver mutations in lung cancer) are more easily targeted by drugs and are less prone to resistance (as seen in clinical cases where EGFR-positive patients treated with drugs such as erlotinib or osimertinib have prolonged progression-free survival). These mutations may affect only a limited number of upstream and downstream molecules and signaling pathways; however, they form the foundation for tumor survival and extensive expansion. In essence, they fundamentally shape the entire tumor. Suppressing these mutations would undoubtedly have a profound, potentially devastating impact on the overall development of the tumor. If this theoretical model holds true, we may observe in gastric cancer models that after STAT3 inhibition, many pathways continue normal operation through compensatory molecules specific to each pathway. Another model, termed the Rapid Evolution and Molecular Tide Interaction Model, suggests further complexities in the interplay between mutations and tumor dynamics, enhancing our understanding of drug resistance and treatment efficacy in cancer therapy. On the basis of the fundamental principles of evolution, we can conceptualize tumor tissue as a population, with each tumor cell as an individual. Each cell possesses a unique molecular phenotype, wherein the roles of widely expressed genes such as STAT3, KRAS, and P53 vary significantly across physiological activities. Moreover, these roles are intricately influenced by the surrounding molecular and immune microenvironments. Consequently, the effectiveness of inhibitors targeting STAT3, KRAS, P53, and others is limited. Furthermore, the expression of these pathways within each cell follows regular patterns on the basis of factors such as the cell cycle and circadian rhythms. For example, pathways upstream and downstream of DNA polymerase during the S phase are broadly activated, whereas pathways related to microtubule proteins are activated during the G2 phase. This molecular tide further increases the diversity and complexity of tumor cell behavior over time, thereby diminishing the efficacy of small molecule targeted inhibitors. The rapid accumulation of genetic mutations within tumor cells, along with strong selective pressures exerted by the body's immune system and therapeutic interventions, leads to rapid tumor evolution and the emergence of alternative pathways. The interaction between molecular tides and rapid evolution accelerates the proliferation of diverse tumor cell phenotypes, making the development of inhibitors that target widely present gene mutations such as STAT3, KRAS, and P53 extremely challenging.

To address the challenges and bottlenecks associated with the use of STAT3 inhibitors in gastric cancer treatment, a theoretically feasible approach involving leveraging the high efficiency and specificity of antigen-antibody interactions could be proposed. It has been demonstrated that monoclonal antibodies targeting the oncostatin M receptor (OSMR), which is involved in STAT3 signaling, can inhibit STAT3 activation and consequently reduce tumor growth in ovarian cancer models [142]. Considering that monoclonal antibodies directly targeting STAT3 and IL-6 have been developed [143,144], future researchers might utilize computer modeling to modify or redesign such antibodies on the basis of their existing structures. These redesigned antibodies could exploit the phenotypic changes induced by STAT3 overexpression, such as the amplification of the IL-6 receptor (IL-6R) [103], gp130 [145], and EGFR [103], to recognize gastric cancer cells accurately and specifically with overexpressed STAT3. The proposed complex antibody could incorporate special domains that bind these specific phenotypes and facilitate endocytosis in tumor cells. Once internalized, the multiple functional sites of an antibody can simultaneously inhibit STAT3 and one or more of its upstream or downstream molecules, thereby enhancing precision and cytotoxic efficacy. To increase the specificity and control of phagocytosis, several sophisticated mechanisms can be incorporated into antibody design:

**Probody Technology**: This approach involves an antibody that possesses a masked binding site. The masking fragment can be cleaved by specific enzymes, enabling activation only under certain conditions, such as in the presence of these enzymes. This ensures that the antibody is selectively activated in specific environments [146].

**Antibody Double Lock Mechanism:** Antibodies are engineered to remain inactive upon binding to a single antigen. They undergo a structural transformation only when they bind simultaneously to a second antigen or more, thereby exposing the Fc segment. This exposed segment can then be recognized by macrophages or other phagocytes, facilitating clearance [147].

**Engineered Fc Segment:** In this design, the Fc segment of the antibody is genetically modified to remain concealed unless multiple antigens are bound. Upon such binding, the Fc segment becomes exposed, making the antibody recognizable to phagocytic cells, thus enabling a targeted immune response [148].

**Induced Aggregation Technology:** Antibodies are designed to undergo conformational changes or to aggregate only when they bind to multiple antigens at the same time. This aggregation exposes the segments that are recognizable by phagocytes, thereby triggering phagocytosis [149].

These targeted strategies improve the therapeutic efficacy and safety of antibodies by reducing off-target effects and enhancing their activity in specific pathological contexts. This multifaceted approach aims to improve the targeting and destruction of STAT3-overexpressing gastric cancer cells by exploiting their unique surface markers and leveraging advanced antibody engineering techniques to maximize therapeutic outcomes. Considering that individualized RNA CD8^+^ T-cell vaccines for pancreatic cancer patients have already entered clinical trials [150], this type of antibody could also be tailored for gastric cancer patients on the basis of the relative levels of STAT3 and its associated signaling molecules within each patient's body, thereby embodying the principle of precision medicine.

## 10 Summary and prospects

This article highlights the role of STAT3 as an important transcription factor that plays a key role in various cellular functions associated with gastric cancer. The signaling landscape upstream of STAT3 consists of several modulators, including noncoding RNAs and small proteins, which control gene expression and epigenetic frameworks either by activating or inhibiting STAT3, thus affecting the development of gastric cancer. However, many of these modulators and their corresponding pathways remain unknown, highlighting the need for further research. Advancements in technology are set to revolutionize the future of healthcare with the generation of large-scale digital data sets through next-generation sequencing (NGS), the application of image processing algorithms, the utilization of patient-related health records, the influx of data from extensive clinical trials, and the predictive modeling of diseases. Oncology remains at the forefront of leveraging AI for comprehensive cancer management. This includes the use of genetic information from patients, encompassing transcriptomics and metabolomics, to enable early detection and tailored treatments [151].

Alternative signaling routes that gastric cancer cells may use when STAT3 is inhibited need to be further investigated. Advancements in research methodologies may lead to the use of big data and AI to evaluate STAT3 and its affiliated signaling molecules in cancerous and noncancerous gastric tissues. Such analyses can reveal the precise, patient-specific selection of STAT3 inhibitors, leading to their synthesis through genetic engineering, which is an innovative therapeutic intervention designed to attenuate the pathological influence of STAT3 while retaining its key physiological functions. The implementation of this strategy can help develop precision medicine, which can increase treatment efficacy and modify side-effect profiles to prolong patient survival and improve patient quality of life.

## Formatting of funding sources

If no funding has been provided for the research, it is recommended to include the following sentence:

This research did not receive any specific grant from funding agencies in the public, commercial, or not-for-profit sectors.

## Ethical statement

This article is written in accordance with all ethical standards.

## CRediT authorship contribution statement

**Muyang Chen:** Writing – review & editing, Writing – original draft, Visualization, Validation, Supervision, Resources, Investigation, Formal analysis, Conceptualization. **Tongshan Wang:** Writing – review & editing, Investigation, Funding acquisition, Conceptualization. **Dianzhe Tian:** Writing – review & editing, Methodology, Formal analysis. **Chaorui Hai:** Writing – review & editing, Visualization, Formal analysis. **Zixuan Qiu:** Writing – review & editing, Funding acquisition.

## Declaration of generative AI and AI-assisted technologies in the writing processes

During the preparation of this work the author(s) used [NAME TOOL/SERVICE] in order to [REASON]. After using this tool/service, the author(s) reviewed and edited the content as needed and take(s) full responsibility for the content of the publication.

## Declaration of competing interest

The authors declare no conflicts of interest.
